# Activating α7nAChR suppresses systemic inflammation by mitigating neuroinflammation of the medullary visceral zone in sepsis in a rat model

**DOI:** 10.1515/tnsci-2022-0345

**Published:** 2024-08-14

**Authors:** Lin Peng, Hongbing Li, Cheng Zhang, Weiwei Jiang

**Affiliations:** Department of Gynecologic Oncology of Hubei Cancer Hospital, Hubei Province, 430079, Wuhan, China; Emergency Department of the First People’s Hospital, Guiyang, Guizhou Province, 550002, China

**Keywords:** sepsis, medullary visceral zone, α7nAChRs, neuroinflammation

## Abstract

Our previous studies have shown that activating α7nAChRs suppresses systemic inflammation and immunity through the cholinergic anti-inflammatory pathway (CAP) in early sepsis. Now that the medullary visceral zone (MVZ) is the center of CAP and responsible for regulating systemic inflammation, what changes will occur in MVZ’s pathology and function in sepsis, especially when interfering with α7nAChRs? Does activation of MVZ’s α7nAChRs contribute to the inhibition of systemic inflammation? To clarify these issues, we explored the systemic inflammation and immunity state by detecting serum levels of TNF-α, IL-6, HMGB1, sCD14, and CD4^+^CD25^+^Treg and TH17 lymphocytes percentage, meanwhile, we analyzed the apoptosis of cholinergic and catecholaminergic neurons and the expressions of tyrosine hydroxylase (TH) and choline acetyltransferase (CHAT) in MVZ in sepsis and the interfering effects on α7nAChRs. In this study, we found that in sepsis, serum TNF-α, IL-6, HMGB1, sCD14, CD4^+^CD25^+^Treg, and TH17 lymphocytes significantly increased and the ratio of Treg/TH17 significantly decreased, cholinergic and catecholaminergic neurons underwent apoptosis with low expressions of TH and CHAT in MVZ; activation of α7nAChRs not only significantly decreased the levels of septic serum TNF-α, IL-6, HMGB1, sCD14, and TH17 lymphocytes (*P* ＜ 0.05), but also significantly reduced cholinergic and catecholaminergic neurons’ apoptosis, and promoted expressions of TH/CHAT. Our study reveals that sepsis undermines MVZ through neuroinflammation which contributes to the uncontrolled systemic inflammation. Activating central α7nAChRs is not only helpful to restore MVZ’s structure and function but also beneficial to subside the inflammatory storm in sepsis. Even if MVZ is damaged in sepsis, cholinergic neurons in MVZ still regulate the systemic inflammation stably.

## 1 Introduction

Sepsis constitutes a serious threat to human health [1], and it represents a dysregulated immune response to infection which may result in fatal organ failure. The uncontrolled inflammatory storm is the key mechanism for the multiple organ dysfunction syndrome and septic shock [2]; it is also associated with intestinal permeability, endothelial injury, blood–brain barrier (BBB) breakdown, and short- and long-term mortality [3]. Growing evidence shows the importance of neuromodulation on the inflammatory cascade [4], therefore restoring the normal neuro-regulation of inflammation and immunity is crucial for sepsis-induced inflammatory storm [5].

**Figure 1:**
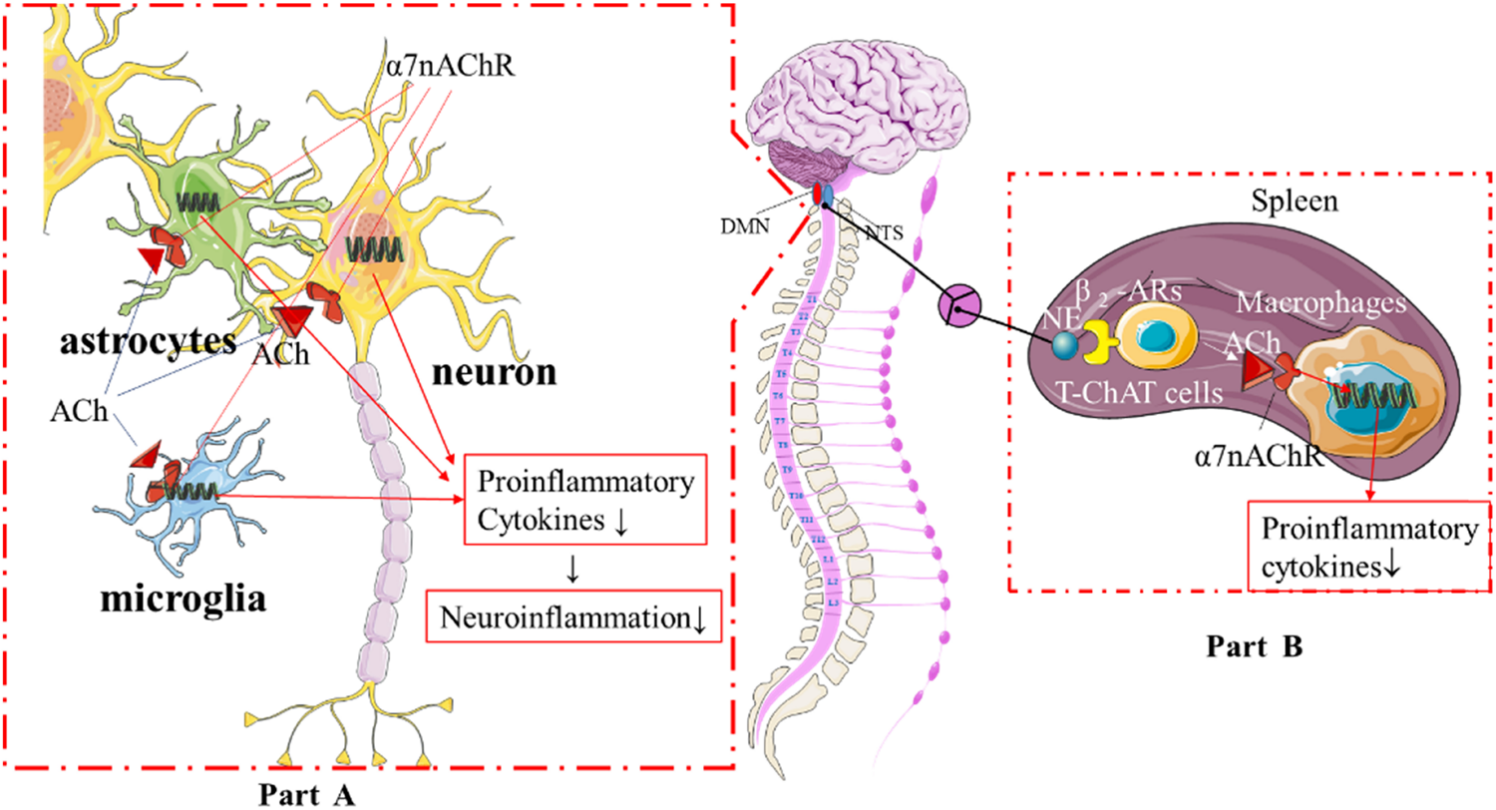
Vagus nerve suppresses systemic inflammation and neuroinflammation through CAP. (a) AChs act on α7nAChRs, widely expressed on the membrane of neurons, microglias, and astrocytes, to suppress the production of proinflammatory cytokines, which dampens the neuroinflammation in MVZ. The normal state of MVZ is essential for the regulation of the systemic inflammation through CAP. (b) CAP modulates systemic inflammation through spleen, which acts as the immune regulatory center. The splenic nerve, innervated by the vagus nerve, releases norepinephrine, which acts on T cells in the spleen and causes the latter to release ACh. ACh acts on α7nAChRs macrophages and inhibits them from releasing pro-inflammatory cytokines so as to modulate the systemic inflammation. By controlling the releasing amount of ACh, CAP can effectively adjust the intensity of systemic inflammation according to the needs of the body.

According to Tracey’s inflammatory reflex theory, which is the most eminent and widely recognized [6], the cholinergic anti-inflammatory pathway (CAP) which belongs to the vagus nerve can quickly sense and efficiently modulate systemic inflammation and immune [7,8]. When CAP is activated, it can rapidly curb systemic inflammatory storm and immune’s activation by releasing acetylcholine (ACh) [9,10] which combines with a7 nicotinic acetylcholine receptor (α7nAChR) on immune cells and inhibits the synthesis and release of inflammatory cytokines [11,12,13]. ACh modifies immune cell functions through α7nAChRs not only in the peripheral immune system but also in the central nervous system (CNS); thus, α7nAChR plays a key role in linking the nervous and immune systems, by controlling the releasing amount of ACh, CAP can effectively adjust the intensity of systemic inflammation according to the needs of the body. It is reasonable to speculate that as the fundamental regulatory center of CAP [14], the medullary visceral zone’s (MVZ) structure and function state play a significant role in the modulation of systemic inflammation and immunity in sepsis (Figure 1b).

In sepsis, MVZ is also subjected to neuroinflammation like other central nervous structures [15]. Neuroinflammation will inevitably affect MVZ’s structure and function, including its modulation of systemic inflammation, but the concrete effect needs to be disclosed. In addition, the widely expressed α7nAChRs not only on astrocytes and microglia but also on neurons were involved with mitigating neuroinflammation and improving neurological function in neurodegenerative diseases [16], stroke [17], and recognitive impairment [18]. Whether activation of α7nAChRs will alter MVZ pathology and further improve systemic inflammation in sepsis remains unknown (Figure 1a). This study conducted a series of experiments to explore the pathological features of MVZ in sepsis, especially when intervention with CAP, so as to search for some therapeutic targets to help to correct the early cytokines storm in sepsis.

## 2 Materials and methods

### 2.1 Animals and experimental model of sepsis

A total of 64 adult, specific pathogen-free, male Sprague-Dawley rats (weighing 250–280 g, 8 weeks old) were purchased from the Experimental Animal Center of Three Gorges University, License Number: SYXK (Hubei) 2018-0104. About five rats were housed in each cage, and they were maintained under specific pathogen-free conditions in the Laboratory Animal Center of the First Hospital of Guiyang. The feeding condition was kept as follows: the temperature was controlled at 21 ± 0.5, a 12 h light–dark cycle (lights on during 07:00–19:00) was set, and standard chow and tap water were provided ad libitum. All rats adapted to the experimental settings for 7 days prior to the treatment.

Sepsis models were prepared by cecal ligation and puncture (CLP) operation [19] under isoflurane inhalation for anesthesia [20]. The CLP operation process is as follows: cut the abdominal cavity about 2 cm to find and isolate the cecum, then ligate about 1/3 of the cecum with a 5–0 suture, and pierce the ligated cecum with a 21-G needle twice. Gently squeeze a small amount of intestinal contents from the puncture hole; at last, return the processed cecum into the abdominal cavity and suture the abdominal cavity. After the CLP operation, the rats’ bodies were curled up and inactive, and they breathed fast and had little and slow response to the outside world. These appearances indicated that the model is successful. Afterward, they were accepted piperacillin (50 mg/kg, qd × 3d) for controlling infection and reducing the mortality and saline (1 mL/100 g, qd × 3d) for fluid resuscitation by intraperitoneal injection [21]. The operation was finished under anesthesia with isoflurane inhalation [22]. All experimental procedures were carried out in compliance with the guidelines of the Institutional Animal Care and Use Committee (IACUC).

### 2.2 Animal grouping and treatment

After a week of adaptive feeding, rats were assigned randomly to five groups, they were separately treated as follows: (1) Control group (eight rats): no interventions were took. (2) Sham group (eight rats): rats were underwent open and suture of the abdominal cavity without CLP. (3) Model group (16 rats): the standard CLP operation as above-mentioned was implemented for each rat. (4) GTS-21 group (16 rats), after CLP operation, rats were accepted intraperitoneal injection of GTS-21, a specific agonist of α7nAChR, which was used as an activator of CAP, referring to the related studies [23,24], the dose and duration are 4 mg/kg, qd × 3d. (5) Methyllycaconitine (MLA) group (16 rats): after CLP operation, rats were accepted intraperitoneal injection of MLA, the antagonist of α7nAChR, which was used as a blocker of CAP, referring to the related study [25], the dose and duration are 4.8 mg/kg, qd × 3d.

### 2.3 Rats’ mortality rate and MSS

Rats’ mortality rates among different groups were recorded and analyzed with Kaplan–Meier survival curve. Moreover, murine sepsis score (MSS) was assessed. MSS was a cumulative point from each rat’s points of appearance, consciousness, behavior, response to stimuli, reaction of opening eyes, breathing frequency, and quality [26]. MSS can be used to judge if or not the model rat develops into sepsis rat and evaluate its severity of sickness. Generally, when a rat’s MSS was more than 4 points, we can think that it is a sepsis rat. In addition, the higher the score, the more serious the rat [27]. Three experimentalists scored every rats separately, and the average points were taken as the MSS of the rat.

### 2.4 Enzyme-linked immunosorbent assay (ELISA)

After 3 days of treatment, there were, respectively, 9, 8, and 11 rats who died in the model group, the GTS-21 group, and the MLA group. All the survival rats were sacrificed to collect blood and medullary tissue for analysis under anesthesia with isoflurane inhalation. For ELISA analysis, 2 mL of blood was taken from the right atrium of the surviving rats under isoflurane inhaling for anesthetization and preserved at room temperature, 2 h later, blood samples were centrifuged at 3,000 rpm for 10 min and the serum was collected for detection. 100 μL of serum were taken to the wells of these kits such as TNF-α (batch number: E-EL-R0019c; Elabscience), IL-6 (batch number: E-EL-R0015c; Elabscience), IL-10 (lot number: E-EL-R0016c; Elabscience), HMGB1 (batch number: E-EL-R0505c; Elabscience), and sCD14 (batch number: CSB-E11178r; Cusabio), respectively, According to the manufacturer’s instructions, after such steps as incubation, washing, reaction with working solution and densitometry, serum contents of TNF-α, IL-6, IL-10, HMGB1, and sCD14 were determined with ELISA.

### 2.5 Flow cytometry (FCM) analysis

For FCM, 2 mL of fresh blood was collected from the right atrium and centrifuged at 400–500×*g* at room temperature for 30 min to collect leukocytes for testing. After repeated centrifugation and dilution, 1 × 10^6^ lymphocytes were collected into flow cytometry tubes and the relevant fluorescent-labeled antibodies were added and incubated, including CD25-PE monoclonal antibody (Invitrogen), lot number: 12-0390-82; CD4-FITC monoclonal antibody (Invitrogen), lot number: 11-0040-82; and IL-17A-PE monoclonal antibody (Invitrogen), lot number: 12-7177-81. At last, after repeated centrifugation and washing three times, the samples were resuspended in 0.2 mL of phosphate buffer solution (PBS) containing 0.5% bovine serum albumin and analyzed by flow cytometry.

### 2.6 Terminal deoxynucleotidyl transferase (TdT)-mediated dUTP nick end labeling (TUNEL)

After 3 days, rats were sacrificed under anesthesia with inhalation of isoflurane, then they were perfused with heparinized saline transcardiac for 60 min, and medulla oblongata was collected for TUNEL and fluorescent immunolabeling analysis.

In the rostral to caudal direction, there were transitional changes in the size, shape, and number of nuclei in the MVZ. From the plane of area postrema (AP) to the obex, the nucleus tractus solitarius (NTS) and vagus dorsal motor nucleus (VDMN) become larger, and NTS becomes wider and divides into multiple subnuclei. To ensure the accuracy and comparability of the results, we specified the exact site where the sections were taken. Combined with the purpose of this study, the MVZ slices were derived from the plane of the AP to the obex. The medulla oblongata was embedded in paraffin and cut into thin slices (30 μm).

TUNEL was used to observe the neuronal apoptosis in MVZ. The sections were dewaxed, gently rinsed with PBS, and immersed in proteinase K solution. Afterwards, samples were infiltrated in 100 μL equilibration buffer, the TdT buffer was added to incubate for 10 min at room temperature, then the sections were washed three times with PBS prior to 4′,6-diamidino-2-phenylindole (DAPI) (produced by Beyotime Biotechnology, batch number: C1002) were added to stain the nuclei of cells, at last fluorescence quencher (produced by Southernbiotech, batch number: 0100-01) were used to seal the sections. Fluorescent microscope (Olympus, BX53, Japan) was used to observe and take photos of these sections. ImageJ software (National Institutes of Health, V1.8.0.112) was used to calculate the apoptosis index (the average ratio of the apoptotic cells to the total cells) [28].

### 2.7 Immunofluorescence

Sections were deparaffinized, antigen repaired and blocked with goat serum (1 h), afterward, the primary antibodies were added to incubate at room temperature, and the primary antibodies include anti-Caspase 3 (produced by Wuhan Sanyan Bio. Co., China. Lot: 66470-2-IG, dilution: 1:50), anti-tyrosine hydroxylase (TH, produced by Wuhan Boster Co., China. Lot: BM4568, dilution: 1:50), or anti-choline acetyltransferase (CHAT, produced by Wuhan Bioss Co., China; lot: bs-2423R, dilution: 1:50). Fifteen hours later, fluorescent-labeled second antibodies were added to incubate at 37°C for 1 h, and fluorescent-labeled second antibodies include FITC-labeled goat anti-rabbit IgG (produced by Wuhan Boster Co., China; lot: BA1105, dilution: 1:100) or Cy3 labeled goat anti-mice IgG (produced by Wuhan Boster Co., China; lot: BA1031, dilution: 1:100). Then, sections were washed four times with PBS for 3 min eACh time, and DAPI was added and incubated in the dark for 5 min to stain nuclei of all cells. Leica TCS SL laser scanning confocal spectral microscope (Leica Microsystems) was used to scan the images, and the latter were analyzed with ImageJ (NIH, USA). Nine MVZ images with 400 folds’ enlargement from three rats of every group were analyzed with Image Pro Plus 6.0 (ipp6.0, Mediacybernetics) software [29] and the average optic densities were acquired.

### 2.8 Statistics

All the survival rats were involved in the laboratory analysis. The data were expressed as mean ± standard deviation (*X̄* ± SD) and were analyzed with the SPSS 19.0 software package (SPSS Inc, Chicago, IL, USA). The homogeneity test of variables by Levene’s test is performed first. Analysis of variance was used to compare the differences between any two groups, and the homogeneous data were determined by the Bonferroni test; otherwise, they were judged by the Tamhane test. *P* < 0.05 was considered statistically significant.


**Ethical approval:** The research related to animals’ use has complied with all the relevant national regulations and institutional policies for the care and use of animals. This research was approved by the Committee on Protection, Welfare and Ethics of Experimental Animals in the First Hospital of Guiyang, Guizhou Pro., China (no. 20190107).

## 3 Results

### 3.1 Mortality and MSS of rats among different groups

**Figure 2:**
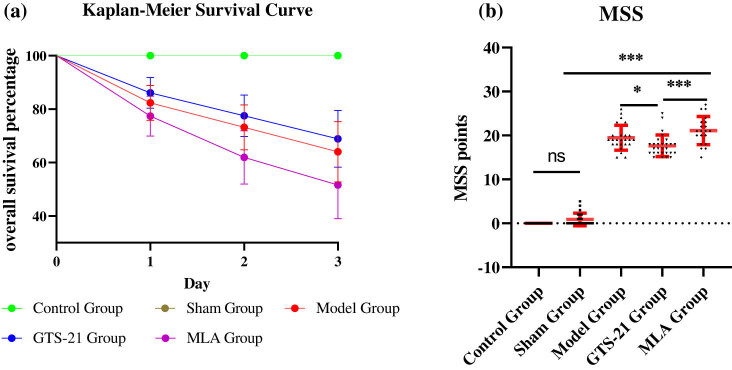
Kaplan–Meier survival curve and MSS among different groups. (a) The Kaplan–Meier survival curve suggests that there was a significant difference in survival percentage among five groups (log rank test: *χ* ^2^ = 15.43, *P <* 0.0001); however, there was no significant difference among the model group, the GTS-21 group, and the MLA group (*χ* ^2^ = 1.21, *P >* 0.05). (b) The scatter plots of MSS show that the average MSS scores of the model group, the GTS-21 group, and the MLA group were much higher than those of rats in the control group. *P < 0.05; ***P ＜ 0.001.

Despite strict disinfection and anti-infection measures being adopted, rats in the septic group still had a high mortality rate. Three days after the operation, there were, respectively, 9, 8, and 11 rats died in the model group, the GTS-21 group, and the MLA group, and the mortality rates were, respectively, 56.3, 50, and 68.8% in these groups. There was no rat died in the control group and the sham group (Figure 2a). The MSS scores of the model group (19.48 ± 2.815), the GTS-21 group (17.64 ± 2.468), and the MLA group (21.10 ± 3.194) were significantly higher than that of the control group (0) and the sham group (0.8750 ± 1.424), all the *P* values <0.0001, GTS-21 obviously reduced the MSS score of sepsis (*P <* 0.05), whereas MLA significantly increased the MSS of the GTS-21 group (*P* < 0.001). In addition, the scores of the same group on different days were not significantly different (Figure 2b).

### 3.2 Inflammatory cytokines levels and α7nAChR intervention

Inflammatory cytokines serum concentration (including TNF-α, IL-6, IL-10, sCD14, and HMGB1) in the model group, the GTS-21 group, and the MLA group were significantly higher than those in the control group and the sham group; for TNF-α, they are, respectively (pg/mL), 127.41 ± 13.23, 91.48 ± 13.18, 146.81 ± 18.61 vs 47.40 ± 8.31; for IL-6, they are, respectively (pg/mL), 99.41 ± 12.70, 81.56 ± 9.49, 108.75 ± 12.57 vs 39.16 ± 7.04; for IL-10, they are, respectively (pg/mL), 90.17 ± 18.77, 66.36 ± 18.55, 114.92 ± 18.61 vs 32.39 ± 7.78; for HMGB1, they are, respectively (pg/mL), 1098.46 ± 143.84, 859.65 ± 127.21, 1250.11 ± 215.91 vs 570.54 ± 101.67; for sCD14, they are, respectively (ng/mL), 367.86 ± 66.21, 290.62 ± 57.43, 481.65 ± 80.72 vs 184.70 ± 45.08, all the *P* values <0.01. All these cytokines show no difference between the control group and the sham operation group.

**Figure 3:**
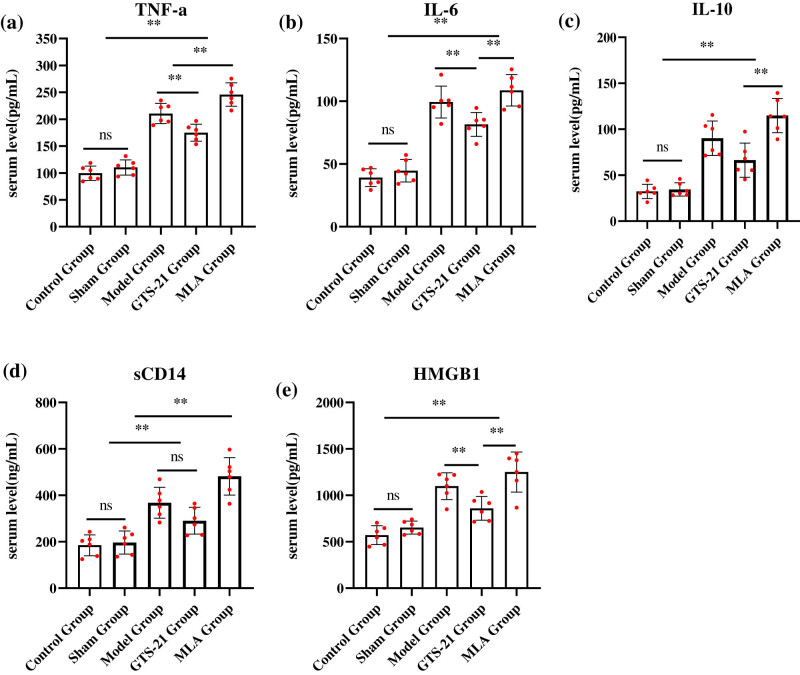
Concentration of serum cytokines among different groups of rats. TNF-α: tumor necrosis factor alpha; IL-6: interleukin 6; IL-10: interleukin 10; sCD14: soluble CD14, precepsin; HMGB-1: high mobility group box-1. (a–c) Serum concentration (pg/mL) of TNF-α, IL-6, and IL-10 among different groups; (d) serum sCD14 concentration (ng/mL) among different groups, usually as an inflammatory marker in early stage of sepsis; (e) serum concentration of HMGB1 (pg/mL), usually as an inflammatory marker in late stage of sepsis; sepsis causes serum level of inflammatory cytokines, including TNF-α, IL-6, IL-10, sCD14, and HMGB1 significantly increased; these cytokines levels in the model group, the GTS-21 group, and the MLA group were significantly higher than those in the control group and the sham group, except IL-10 and sCD14, GTS-21 significantly decreased the other cytokines’ level; all inflammatory cytokines levels reached the highest in the MLA group, and there were significant differences when compared to the GST-21 groups. *P < 0.05; **P < 0.01.

Besides IL-10 and sCD14, serum concentrations of TNF-α, IL-6, and HMGB1 in the GTS-21 group were significantly lower than those in the model group; they are, respectively: 174.86 ± 15.66 vs 210.56 ± 18.76; 91.48 ± 13.18 vs 127.41 ± 13.23; and 859.65 ± 127.21 vs 1098.46 ± 143.84, all the *P* values <0.01; all inflammatory cytokines levels reached the highest in the MLA group, and there were significant differences when compared to the GST-21 groups (*P* < 0.01 or *P* < 0.05) (Figure 3).

### 3.3 Immunity and α7nAChR intervention

**Figure 4:**
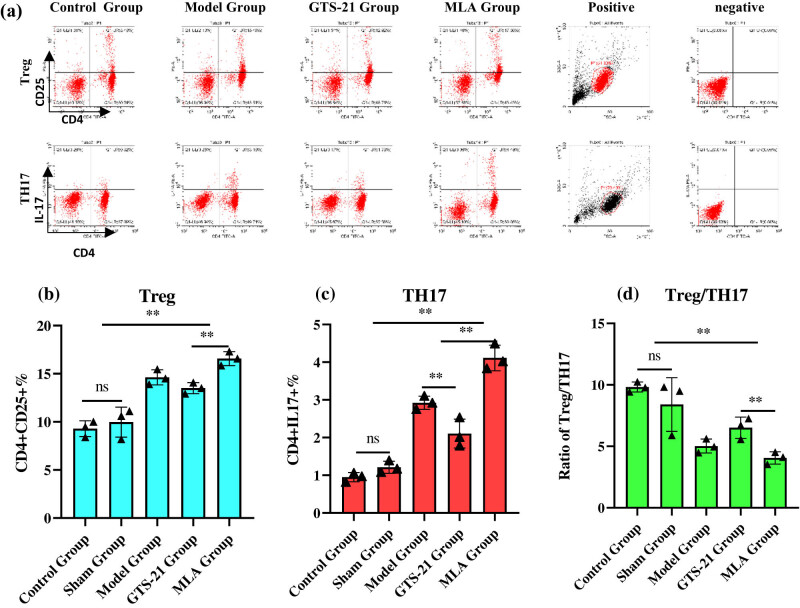
Blood lymphocyte percentages of Treg and TH17 among different groups. (a) Representative scatter plot images of CD4^+^CD25^+^Treg and CD4^+^IL-17^+^TH17 lymphocyte of different groups. (b) Histogram of double positive pencentage of CD4^+^CD25^+^Treg lymphocytes among different groups. (c) Histogram of double positive pencentage of CD4^+^IL-17^+^TH17 lymphocytes among different groups. (d) Histogram of the ratio of Treg/TH17 among different groups. Sepsis causes a significantly increase of Treg and TH17 lymphocytes and signifies activation of innate and acquired immunity. GTS-21 obviously decreases these two kinds of lymphocytes, especially TH17 lymphocyte percent, suggesting activation of CAP curbs the immunity, whereas MLA’s effect is the opposite, denoting that blocking CAP exaggerates the immunity. It can also be seen from the ratio of Treg/TH17. *P < 0.05; **P < 0.01.

In the model group, the GTS-21 group, and the MLA group, blood lymphocytes percentages of CD4^+^CD25^+^Treg and CD4^+^IL17^+^TH17 were significantly higher than those in the control group and the sham group, they were, respectively (%), 14.62 ± 0.78, 13.50 ± 0.58 and 16.57 ± 0.73 vs 9.30 ± 0.82; 2.92 ± 0.18, 2.11 ± 0.38 and 4.11 ± 0.34 vs 0.95 ± 0.12, all the *P* values <0.01. The ratios of Treg/TH17 in the model group, the GTS-21 group, and the MLA group were significantly lower than that in the control group; they were, respectively, 5.02 ± 0.57, 6.51 ± 0.86, and 4.06 ± 0.50 vs 9.82 ± 0.41, *P* < 0.05 or 0.01. GTS-21 evidently reduced the percentage of TH17 lymphocyte (2.11 ± 0.38 vs 2.92 ± 0.18, *P* = 0.02), but it neither reduced Treg lymphocyte percentage nor increased the ratio of Treg/TH17 significantly in sepsis, (13.50 ± 0.58 vs 14.62 ± 0.78, *P* = 0.62; 5.02 ± 0.57 vs 6.51 ± 0.86, *P* = 0.51); the percentages of Treg and TH17 lymphocytes in the MLA group were significantly higher than those in the GTS-21 group (*P* < 0.05 or 0.01) (Figure 4).

### 3.4 The pathological features of MVZ

TNUNEL analysis shows that compared to the control and sham groups, there were much more apoptotic neurons in the septic groups, especially in the MLA group and the model group. GTS-21 had the tendency to reduce apoptosis of MVZ neurons in septic rats.

**Figure 5:**
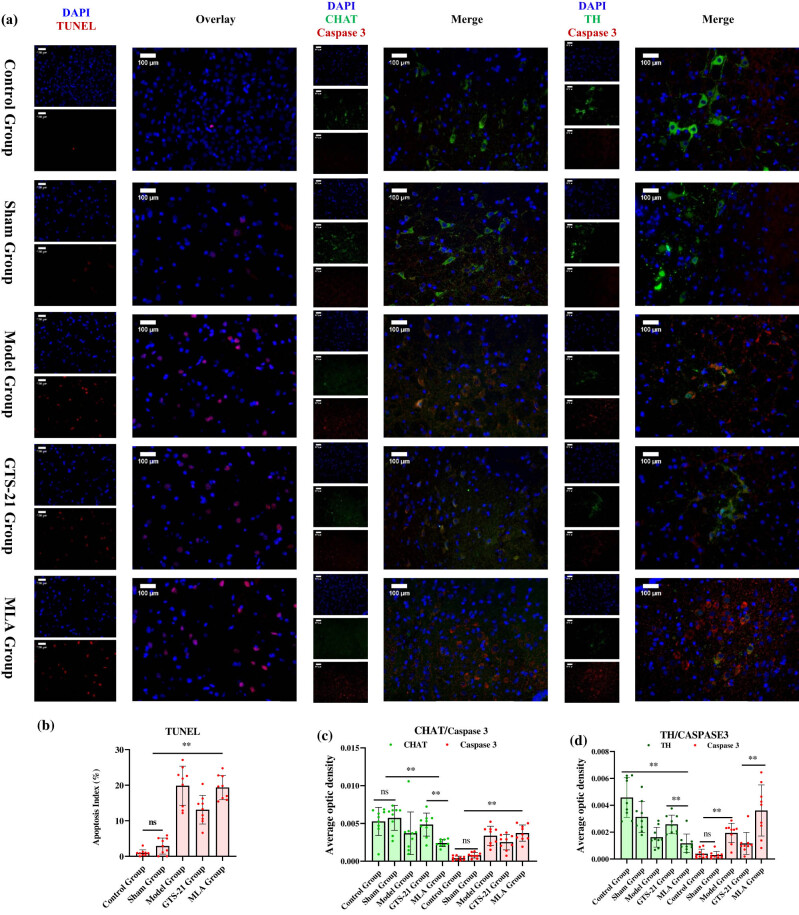
Pathological study of MVZ in sepsis and intervened by CAP (TUNEL and double immunofluorescence-labeling experiments). Figure 4 shows that sepsis induces MVZ neurons (including cholinergic neurons and catecholaminergic neurons) apoptosis and inactiveness. (a) TUNEL and double immunofluorescence labeling images of MVZ. The red fluorescence in the TUNEL column indicates apoptotic cells. In the TH/Caspase 3 column, the green fluorescence indicates the expression of TH, and the red fluorescence indicates the expression of Caspase 3 in the catecholaminergic neurons. In the CHAT/Caspase 3 column, the green fluorescence indicates the expression of CHAT, and the red fluorescence shows the expression of Caspase 3 in the cholinergic neurons. From these images, we can see that sepsis not only causes significant apoptosis of catecholaminergic and cholinergic neurons in MVZ but also affects their activeness. Intervention with CAP can affect the activity and apoptosis of functional neurons in the MVZ area: α7nAChR agonists GTS-21 have the tendency to prevent the cholinergic and catecholaminergic neurons from apoptosis and inactiveness in MVZ, while α7nAChR antagonist MLA significantly accelerates apoptosis and inactiveness of these two kinds of neurons. (b) The histogram of TUNEL analysis, which confirms that sepsis-induced significant apoptosis in MVZ, activating CAP had the tendency to reverse it; on the contrary, blocking CAP exacerbated the apoptosis. (c) The histogram of TH/Caspase 3 expressions in MVZ, which suggests that sepsis causes significant apoptosis of catecholaminergic neurons in MVZ; GTS-21 can reverse the situation, whereas MLA is the opposite; in addition, both sepsis and even sham operation cause significantly low expression of TH, especially when rats were treated by MLA. (d) The histogram of CHAT/Caspase 3 expressions in MVZ, which shows that even though sepsis caused significant apoptosis of cholinergic neurons in MVZ; it did not induce the significantly reduced expression of CHAT except in the MLA group.

From fluorescence double-labeling analysis, the expression of TH obviously decreased in other four groups compared to the control group, and GTS-21 had the tendency to up-regulate it; on the contrary, MLA furtherly reduced it remarkably, whereas the expression of CHAT did not decrease significantly in sepsis except in the MLA group. The expression of Caspase 3 was evidently up-regulated in three septic groups when compared to the control group and the sham group, GTS-21 had the tendency to curb its expression, and MLA boosted its expression (Figure 5).

## 4 Discussion

Based on our previous studies, this study gives us some meaningful hints, we can reasonably speculate that systemic inflammatory storm may be correlated to the damaged MVZ in sepsis; what is more, activation of α7nAChRs suppresses systemic inflammation by mitigating neuroinflammation of MVZ in Sepsis.

In spite of administration of antibiotics is in favor of controlling infection and reducing the mortality of rats, it may affect the occurrence of sepsis. In our experiment, despite the administration of piperacillin, there are heavy mortality rates in the CLP rats. To compare the results among groups, except rats in the control group, all the other rats accepted piperacillin by intraperitoneal injection after operation. Additionally, we have confirmed that intraperitoneal injection of piperacillin did not stop the occurrence of sepsis in the CLP rats according to our experiment results, especially the serum cytokines level.

To testify CAP’s function on systemic and MVZ’s inflammation, we choose the agonist and antagonist of α7nAChR in this study. GTS-21, an α7nAChR-specific agonist, can bind and activate α7nAChR just as ACh, to exert α7nAChR mediated anti-inflammatory effects [30], whereas methyllycaconitine (MLA), an α7nAChR-specific antagonist, can bind and block α7nAChR, to prompt LPS-induced release of TNF-α [31,32]. According to the previous study, we selected GTS-21 (4 mg/kg) and MLA (4.8 mg/kg) to activate or block α7nAChR. We can see that GST-21 significantly reduces serum levels of inflammatory mediators such as TNF-α, IL-6, IL-10, sCD14, and HMGBl and the percentage of TH17 lymphocytes in sepsis, whereas MLA-induced increases in serum cytokine levels, and a decrease in the ratio of Treg/TH17 suggests that blocking CAP leads to intensify the systemic inflammation. In other words, the activation of CAP significantly mitigates the inflammatory storm caused by sepsis.

α7nAChR is a key receptor that mediates between the CNS and immune systems, and activation of α7nAChR in macrophages reduces inflammatory cytokine secretion and regulates apoptosis, proliferation, and polarization of macrophage. Administration of a specific α7nAChR agonist (PNU 282,987) reduces the infiltration of inflammatory M1 macrophages into the inflammatory area and increases the recruitment of M2 anti-inflammatory macrophages to the inflammatory area [33]. Moreover, *in vitro* studies have shown that GTS-21 suppresses M1 macrophage polarization while promoting M2 macrophage polarization through the upregulation of TNF-α/PI3K and phosphorylation of STAT3 [34,35]; through polarization of macrophage, activation of α7nAChR eventually reduces the systemic inflammatory level in sepsis [9]. Moreover, α7nAChR activation prevents almost all the NF-κB-mediated pro-inflammatory pathways, for example, it inhibits the expression of TLR4 and CD14 [36] via α7nAChR/PI3K signaling pathway [37], it also inhibits ATP-mediated IL-1β release in human and rat monocytes [38], and in addition, it inhibits the production of cytokines from monocytes activated by ligands for TLR2, TLR3, TLR4, TLR9, and RAGE [39]. In our study, we confirmed that activation of α7nAChRs, or potentiate CAP is a significant measure to curb the systemic inflammation in sepsis; on the contrary, blocking α7nAChRs, or suppressing CAP leads to exacerbate the systemic inflammation.

Except for the direct effect on the peripheral immune cells, whether CAP is involved with a central effect in the modulation of systemic inflammation remains unclear. After all, GTS-21 and MLA can pass through the BBB, they may also act on the central α7nAChRs, which are also expressed on the membrane of microglia and astrocytes and can significantly inhibit neuroinflammation after activation [40,41]. Study has confirmed that activation of cholinesterase inhibitors (ChEIs) that can pass through BBB showed greater anti-inflammatory efficacy than those that cannot [42]; it suggests that activation of central α7nAChRs is a valuable and considerable way for the uncontrollable systemic inflammatory storm in early sepsis. The underlying mechanism needs to be clarified.

MVZ is mainly composed of the NTS, rostral ventrolateral medulla (RVLM), and VDMN; it bridges not only the sympathetic and parasympathetic systems but also the autonomic nervous and the hypothalamic–pituitary–adrenal axis, and still the afferent and efferent systems of vagus nerve to coordinate the regulation of systemic inflammation [4]. Therefore, MVZ is the key center of CAP. What effects will MVZ’s neuroinflammation cause on CAP’s function and interfered with by α7nAChRs?

In this study, as shown in previous studies, sepsis induces neuroinflammation in MVZ [43,44].

From TUNEL and fluorescence double-labeling analysis, the up-regulated expressions of Caspase 3 signified that both cholinergic and catecholaminergic neurons had gone through evident apoptosis in sepsis. The activation of α7nAChRs significantly reduced cholinergic and catecholaminergic neurons’ apoptosis and promoted expressions of TH/CHAT. The blockage of α7nAChRs is the opposite. Based on the CAP’s inflammatory modifying theory, we can deduce that the suppression of systemic inflammation by GTS-21 should be partly attributed to its central effect by facilitating restoration of the “inflammatory” MVZ; similarly, the exacerbation of systemic inflammation by MLA should be partly attributed to its central effect by aggravating the “inflammatory” MVZ.

Numerous studies show that activation of α7nAChRs alleviates neuroinflammation through multiple ways. For example, it can reduce the production of pro-inflammatory cytokines including IL-17, IFN-γ, and IL-6, as well as increase the production of the anti-inflammatory cytokine IL-10 in encephalitogenic T cells [45]. It can dampen microglia transform into M1 phenotype to inhibit LPS-induced IL-1β and IL-6 elevation, facilitate microglia transform into M2 phenotype, and promote IL-4 and IL-10 production [46]; what is more, activation of α7nAChRs inhibits the cultured microglial proliferation and increases microglial death to reduce M1 monocyte proportions [47,48] and facilitates M2 macrophages survival via STAT3 pro-survival pathway [49]. It also decreases the rate of LPS-induced macrophages migration [50]. Infected α7nAChR−/− mice showed significantly higher expression of pro-inflammatory cytokines such as TNF-α, IL-6, and MCP-1 in the brain [51]. Central activation of the CAP, triggered by central or peripheral administration of α7nAChRs’ agonist, will elicit an anti-inflammatory effect mediated by the CAP and α7nAChRs [52,53]. Our study confirmed that pharmacological activation of CAP can modify the MVZ’s inflammation and function in sepsis.

The evidently reduced expression of TH in the septic group suggests the “suppression” of catecholaminergic neurons in sepsis. GTS-21 has the tendency to prompt the expression of TH, whereas MLA significantly down-regulates it compared to GTS-21, which hints that activating CAP can protect the catecholaminergic neurons and recover their function. However, the expression of CHAT remained unchanged statistically in the septic groups except the MLA group, probably because cholinergic neurons were excited by the sensation of inflammation to synthesize ACh and transmit instructions in sepsis unless they were severely damaged in the MLA group.

Cholinergic neurons in VDMN negatively regulate innate immunity through CAP [54]. Therefore, the apoptosis or inactiveness of catecholamine and cholinergic neurons in MVZ will inevitably affect the modulation of systemic inflammation and immunity, the vitality of cholinergic neurons is directly related to the modulation of systemic inflammation through CAP. Catecholamine neurons are rich in RVLM, and the vitality of catecholamine neurons has an important indirect influence on the modulation of systemic inflammation through CAP. From these studies, we can conclude that cholinergic neurons remain function stable in sepsis which is critical for the modulation of systemic inflammation.

Now that cholinergic neurons of MVZ still stably regulate systemic inflammation, even though they were severely damaged in sepsis, the exact mechanism is unclear but worth scrutinizing. We know that our brain has a wealth of compensatory mechanisms [55,56] employed by neurons and circuits to keep the systemic inflammatory and immune stable [57–59]. For example, in patients and models of Alzheimer’s disease (AD), the amount of nAChRs and cholinergic neurotransmission fluctuate at different stages [60] to maintain stable output of instructions according to the needs of the body. Therefore, complex neural network circuits and synaptic plasticity may be involved, and the concrete mechanism should be explored in the future. In addition, pharmacological acting on α7nAChR may become a therapeutic strategy for sepsis.

This study has certain limitations. The interfering measures only involved a single dose such as GTS-21 (4 mg/kg) and MLA (4.8 mg/kg), a single time point; therefore, the results may be inconclusive. In addition, it only focused on CAP’s modulation of inflammation and immunity and did not consider the sympathetic nerve’s influence. In fact, inflammation is involved in the overactivity of hypothalamic paraventricular nucleus neurons converging on RVLM, thereby triggering sympathetic excitation [61]. In addition, NTS is interconnected between RVLM and locus coeruleus, which control spinal cord-derived catecholaminergic output and will affect the strength of systemic inflammation. Additionally, this study is an animal experiment, clinical studies may be different and need to be further explored in clinical practice.

## 5 Conclusions

In summary, this study shows that systemic inflammation storm caused by sepsis may be correlated to the dysregulation of MVZ. The pharmacological modulation of CAP modifies MVZ neurons’ apoptosis and probably furtherly influences the systemic inflammation. Even if MVZ is damaged in sepsis, the vitality of cholinergic neurons remains stable to maintain a certain level of modulation on systemic inflammation. The further study should focus on how MVZ’s neuroinflammation causes an inhibitory effect on CAP, for example, whether the inflammatory demyelination and the damage of neural plasticity are involved in this pathology is needed for exploration, and the corresponding interfering measures should be searched out to combat sepsis.
